# Inhibition of connexin 43 prevents trauma-induced heterotopic ossification

**DOI:** 10.1038/srep37184

**Published:** 2016-11-16

**Authors:** Bing Tu, Shen Liu, Guangwang Liu, Zhiwei Li, Yangbai Sun, Cunyi Fan

**Affiliations:** 1 Department of Orthopaedic Surgery, Shanghai Jiaotong University Affiliated Sixth People’s Hospital, No. 600 Yishan Road, Shanghai, 200233, China; 2 Department of Orthopaedic Surgery, the Central Hospital of Xuzhou, Xuzhou Clinical School of Xuzhou Medical College, Xuzhou Hospital (affiliated with Medical College of Southeast University), Jiangsu, 221009, China

## Abstract

Heterotopic ossification (HO) can result from traumatic injury, surgery or genetic diseases. Here, we demonstrate that overexpression of connexin 43 (Cx43) is critical for the development and recurrence of traumatic HO in patients. Inhibition of Cx43 by shRNA substantially suppressed the osteogenic differentiation of MC-3T3 cells and the expression of osteogenic genes. We employed a tenotomy mouse model to explore the hypothesis that Cx43 is vital to the development of HO. Inhibition of Cx43 by a specific shRNA decreased extraskeletal bone formation *in vivo*. In addition, we demonstrated that ERK signaling activated by Cx43 plays an important role in promoting HO. ERK signaling was highly activated in HO tissue collected from patient and mouse models. Importantly, de novo soft tissue HO was significantly attenuated in mice treated with U0126. Inhibition of Cx43 and ERK led to decreased expressions of Runx2, BSP and Col-1 *in vivo* and *in vitro*. Moreover, HO patients with low Cx43 expression or ERK activation had a lower risk of recurrence after the lesions were surgically removed. Our findings indicate that Cx43 promotes trauma-induced HO formation by activating the ERK pathway and enhances the expression of osteogenic markers.

Heterotopic ossification (HO), which is commonly observed in cases of traumatic injury, severe burns and invasive surgeries, is the aberrant formation of extraskeletal bone in soft tissues such as muscles, tendons, and ligaments^1^. Traumatic HO occurs in response to injuries such as fractures and fracture-dislocations of joints. Treatment options for HO are limited because the excess bone growth often recurs following surgical resection, and some patients may have HO that is nonresectable because of its location^2^. For several years, the genetic basis of HO has been known to be similar to that of fibrodysplasia ossificans progressiva (FOP), however, the mechanism of HO development is still unclear. The pathophysiology of HO closely resembles the physiologic process of fracture healing^3^. In the case of HO formation, the osteogenesis process is thought to arise from the disordered differentiation and proliferation of precursor cells. Previous findings have shown that progenitor cells obtained from traumatized muscle tissue, which is prone to developing HO, are similar to bone marrow-derived mesenchymal stem cells (MSCs) in morphology and biological function^4^,^5^.

A number of research groups have reported several types of evidence showing that the gap junction protein connexin 43 (Cx43, also called gap junction protein A1, GJA1) controls bone function and development^6^,^7^. Cx43, a gap junction channel protein that mediates the communication between adjacent cells or between a cell and its extracellular environment, is observed to be upregulated under fracture conditions^8^. In addition, fracture healing is impaired in Cx43-deficient mice, which is consistent with delayed osteoblast differentiation and bone formation^9^. These observations suggest that Cx43 facilitates new bone formation. Overexpression of Cx43 in osteoblasts promotes the transcriptional activity of Runx2, and Cx43 knockdown suppresses Runx2-dependent transcription^10^. The signaling molecules mediating the action of Cx43 on osteogenesis include the protein kinase C (PKC) family member PKCδ and the extracellular signal regulated kinases (ERKs), two proteins that were previously reported to regulate Runx2 activity in osteoblasts^11^,^12^. Given the correlations between Cx43 and bone formation, we hypothesized that Cx43 may enhance the development of traumatic HO by interacting with ERK signaling.

In this study, we demonstrated that Cx43 contributes to the development of traumatic HO. Cx43 knockdown substantially attenuated the expression of osteogenic marker genes and markedly inhibited the growth of HO in a mouse model. Cx43 promoted osteogenesis through the ERK pathway. Inhibition of ERK also resulted in decreased HO development. Moreover, traumatic HO patients with low Cx43 expression and ERK activation showed decreased recurrence after surgical removal of the lesions. The data in the present study may provide potential therapeutic target for traumatic heterotopic ossification.

## Results

### Expression of Cx43 is increased in human heterotopic ossification tissues

**Figure 1:**
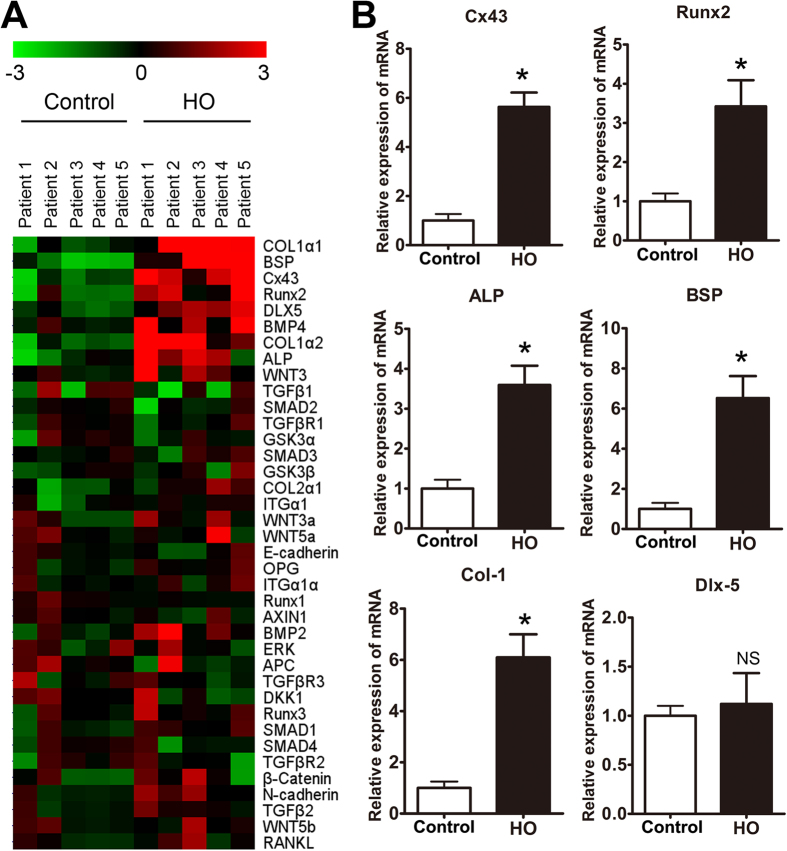
Expression of Cx43 and osteogenic marker genes was enhanced in HO. (**A**) Heat map depicting the relatedness of the gene expression profiles of normal bones and HO. Hierarchical clustering was applied to microarray data, and selected portions of the clustering heat map are presented. Red and green indicate high and low gene expression levels, respectively. (**B**) Total RNA was extracted from normal bones and HO, and the expression of Cx43, Runx2, ALP, BSP, Col-1 and Dlx5 mRNA was detected by real-time PCR. (t-test, n = 5, *p < 0.05; NS, not significant).

To investigate the genes that may be involved in the regulation of HO formation, we used genomic data from 5 patients with elbow HO to compare with data from the control bones, which were collected from 5 traumatic amputation patients. In particular, we noted upregulation of Cx43 and osteogenic marker genes in the heterotopic ossification samples (Fig. 1A). Previous reports have underscored the importance of Cx43 in osteoblast differentiation and skeletal development^13^. Next, the expression levels of Cx43 and osteogenesis-related genes were further confirmed by real-time PCR. We verified that the expression of Cx43 and osteogenic markers, including Runx2, alkaline phosphatase (ALP), bone sialoprotein (BSP) and collagen-1 (Col-1), were increased in HO patients compared with control patients (Fig. 1B). However, no significant difference in Dlx-5 expression was observed between the two groups. Furthermore, soft tissues (joint capsule and ligament) around the HO or near the normal joint (used as normal control) were collected from the HO or traumatic amputation patients. We observed an elevated expression of Cx43, IL-1, IL-6 and IL-8 in the soft tissue surrounding the HO (Supplementary Fig. 1).

### Cx43 promotes osteoblast differentiation by activating the ERK pathway

**Figure 2:**
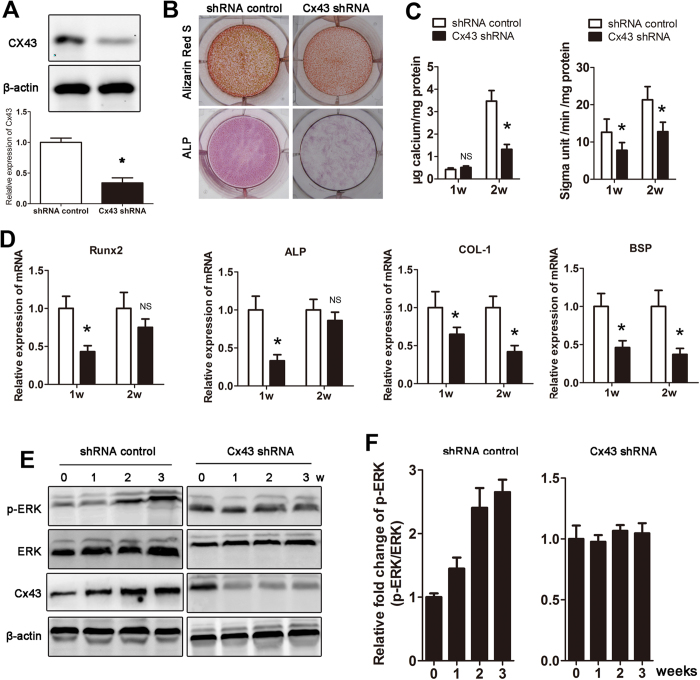
Cx43 promotes osteoblast differentiation by activating the ERK pathway. (**A**) Small hairpin RNAs (shRNA) were used to knock down Cx43 expression in MC-3T3 cells, and a scrambled sequence was used as a control. The expression of Cx43 was detected by Western blot. (**B**) MC-3T3 cells transfected with Cx43 shRNA were induced in osteogenic medium. Alizarin Red S (week 2) and ALP (week 1) staining was performed. (**C**) Calcium deposits and ALP activity were quantified. (**D**) mRNA expression of osteogenic marker genes was detected by real-time PCR at week 1 and 2. (**E**) ERK pathway activation and Cx43 expression were examined weekly by Western blot. (**F**) A densitometric analysis of p-ERK expression was performed (t-test, n = 3, *p < 0.05; NS, not significant).

Aberrant osteogenesis plays an important role in the formation of HO. To determine the effect of Cx43 on osteoblast differentiation, expression of Cx43 was inhibited by a Cx43-specific shRNA transfection. The Cx43 shRNA transfection did not affect the viability of MC-3T3 and C2C12 cells (Supplementary Fig. 2). The inhibitory effect was confirmed by western blot (Fig. 2A). Then, MC-3T3 cells transected with Cx43 shRNA were exposed to osteogenic differentiation medium for 2 weeks. We observed that both calcium deposition and ALP activity were decreased in the Cx43 knockdown group (Fig. 2B,C). Furthermore, our real-time PCR results showed that Cx43 knockdown inhibited osteogenic marker gene expression (Fig. 2D). A similar result was observed in mouse mesenchymal stem cells (Supplementary Fig. 3A–D) Previous studies have suggested that ERK is part of an important downstream pathway of Cx43. We then examined whether the ERK pathway played a role in Cx43-induced osteogenesis. Our data indicated that ERK signaling was activated during the osteogenesis process, meanwhile expression of Cx43 was increased during osteogenesis. However, when Cx43 was knocked down by shRNA, p-ERK expression was maintained at a steady-state during osteogenesis (Fig. 2E,F). To exclude the effects of osteogenic medium on ERK activation, MC-3T3 cells were incubated in α-MEM without osteogenic induction, and Cx43 was knocked down by shRNA. We observed that activation of the ERK and PKC pathway was inhibited at week 1–3 (Supplementary Fig. 4A,B). However, inhibition of ERK by U0126 or shRNA had no effect on expression of Cx43 (Supplementary Fig. 4C).

### Inhibition of Cx43 limits HO formation *in vivo*

**Figure 3:**
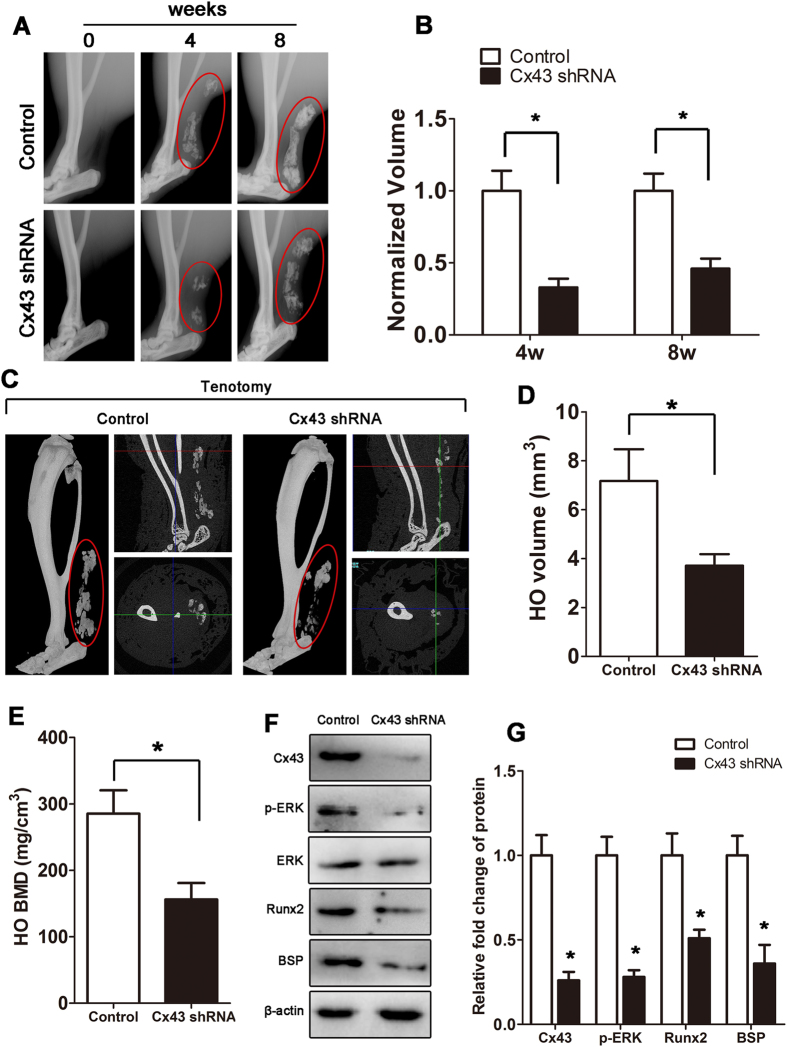
Inhibition of Cx43 limits HO formation *in vivo*. Tenotomy mice were injected with Cx43 shRNA or scrambled RNA weekly at the lesion site. (**A**) HO was visualized by a weekly radiograph. (**B**) The HO volumes were quantified. (**C**) At week 8, a three-dimensional reconstruction (Left), cross-section (Right upper) and sagittal section (Right below) of microCT scans of Cx43 shRNA- and control RNA-treated tenotomy mice were obtained. The red, green and blue lines represent the cross, sagittal and coronal plane in other sections, respectively. (**D**) The HO volumes were quantified. (**E**) The bone mineral density (BMD) of HO was qualified. (**F**) Expression of Cx43, p-ERK, ERK, Runx2, BSP and β-actin was examined by Western blot. (**G**) A densitometric analysis of Cx43, p-ERK, Runx2 and BSP protein expression was performed. (t-test, n = 3, *p < 0.05).

Next, we tested the hypothesis that Cx43 inhibition can prevent HO. For this purpose, mice received a tenotomy were subsequently treated with an injection of Cx43 shRNA at the lesion site. We observed a reduced amount of early endochondral ossification in mice treated with Cx43 shRNA at 3 weeks (Supplementary Fig. 5). Then, the injured limbs were examined by x-ray. The tenotomy mice treated with Cx43 shRNA injection demonstrated a reduction in total HO volume at 4 and 8 weeks after injury (Fig. 3A,B). Consistent with the radiologic data, representative images of 3D μCT reconstructions of a tenotomy mouse showed markedly decreased HO volume after Cx43 shRNA injection (Fig. 3C,D). Moreover, the bone mineral density (BMD) of HO was decreased after the Cx43 shRNA treatment (Fig. 3E). Tissues from the tenotomy site 8 weeks after injury showed diminished levels of Cx43, pERK and osteogenic marker genes, including the early osteogenic gene Runx2 and the later gene BSP (Fig. 3F,G). To test whether Cx43 could inhibit the developed HO, tenotomy mice were maintained for 8 weeks and then treated with Cx43 shRNA injection for another 4 weeks. The x-ray images showed no difference between the two groups at week 12 (Supplementary Fig. 6).

### ERK activation is essential for Cx43-induced HO

**Figure 4:**
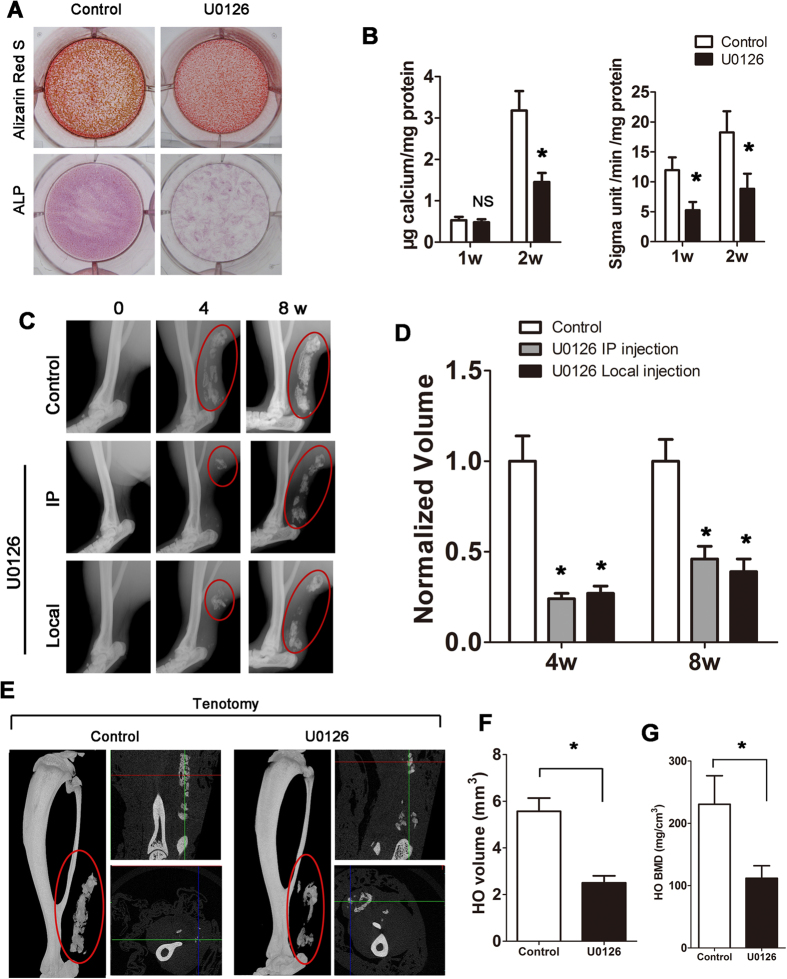
ERK activation is essential for Cx43-induced HO. (**A**) MC-3T3 cells were induced in osteogenic medium and exposed to U0126 for 2 weeks. Calcium deposits were detected by Alizarin Red S at week 2, and ALP staining was performed at week 1. (**B**) Calcium deposits and ALP activity was quantified. (**C**) Tenotomy mice were treated by intraperitoneal or local injection of U0126 every 3 days. Local injection of DMSO was served as the control. HO was visualized by a weekly radiograph (One-way ANOVA, n = 5, *p < 0.05 vs the control; no significant difference was observed between the intraperitoneal and local injection groups). (**D**) The HO volumes were quantified. (**E**) At week 8, three-dimensional reconstruction (Left), cross-section (Right upper) and sagittal section (Right below) of microCT scans of the tenotomy mice. The red, green and blue lines represent the cross, sagittal and coronal plane in other sections, respectively. (**F**) The HO volumes were quantified. (**G**) The bone mineral density (BMD) of HO was qualified. (t- test, n = 3, *p < 0.05).

To investigate whether inhibition of the ERK pathway would limit HO, MC-3T3 cells exposed to U0126 were incubated in osteogenic medium for 2 weeks *in vitro*. When ERK signaling was inhibited by U0126, Alizarin Red S staining showed less calcium deposition (2 weeks) and less ALP activity (1 week) (Fig. 4A,B). Similarly, inhibition of ERK signaling by a specific shRNA suppressed osteogenic differentiation (Supplementary Fig. 7A,B). Then, tenotomy mice were treated with an injection of U0126 at the lesion sites or intraperitoneal injection (IP). Both injection methods decreased the HO formation, while no difference was observed between the local and intraperitoneal injection groups (Fig. 4C,D). As observed in the mice that received the Cx43 shRNA injection, the 3D μCT reconstruction images showed a decreased HO volume after the U0126 local injection (Fig. 4E,F). We observed a decreased BMD in the HO after the Cx43 shRNA injection (Fig. 4G).

### Cx43 enhances the expression of osteogenic markers through ERK

**Figure 5:**
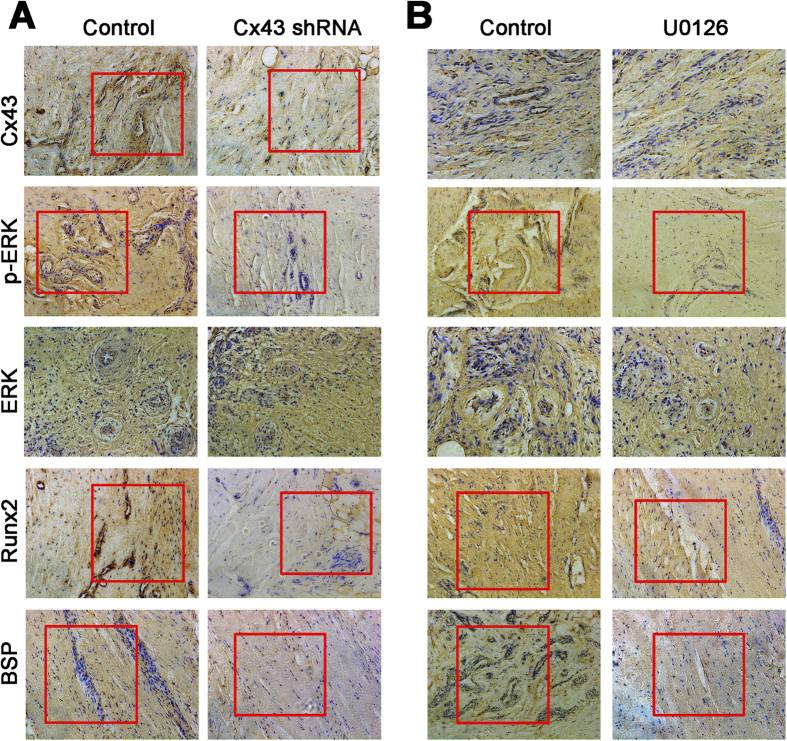
Inhibition of Cx43 and ERK signaling decreases osteogenic marker gene expression *in vivo*. (**A**) Tenotomy mice were treated by local injection of Cx43 shRNA or intraperitoneal injection of U0126. HO tissues were decalcified, and expression of Cx43, p-ERK, ERK, Runx2 and BSP was detected by immunohistochemistry at week 8. (**B**) Cx43, p-ERK, Runx2 and BSP expression in the HO tissues was quantified.

Then, the HO samples from the mice were collected and demineralized. The immunohistochemistry results demonstrated that a Cx43 shRNA injection caused decreased expression of Cx43, p-ERK, BSP and Col-1. Inhibition of ERK signaling by U0126 only decreased the expression of p-ERK, BSP and Col-1, while no change in the Cx43 expression was observed. These data suggest that Cx43 enhances the expression of osteogenic markers by activating ERK (Fig. 5A,B).

### Cx43/ERK signaling is critical for clinical outcomes in HO patients

**Figure 6:**
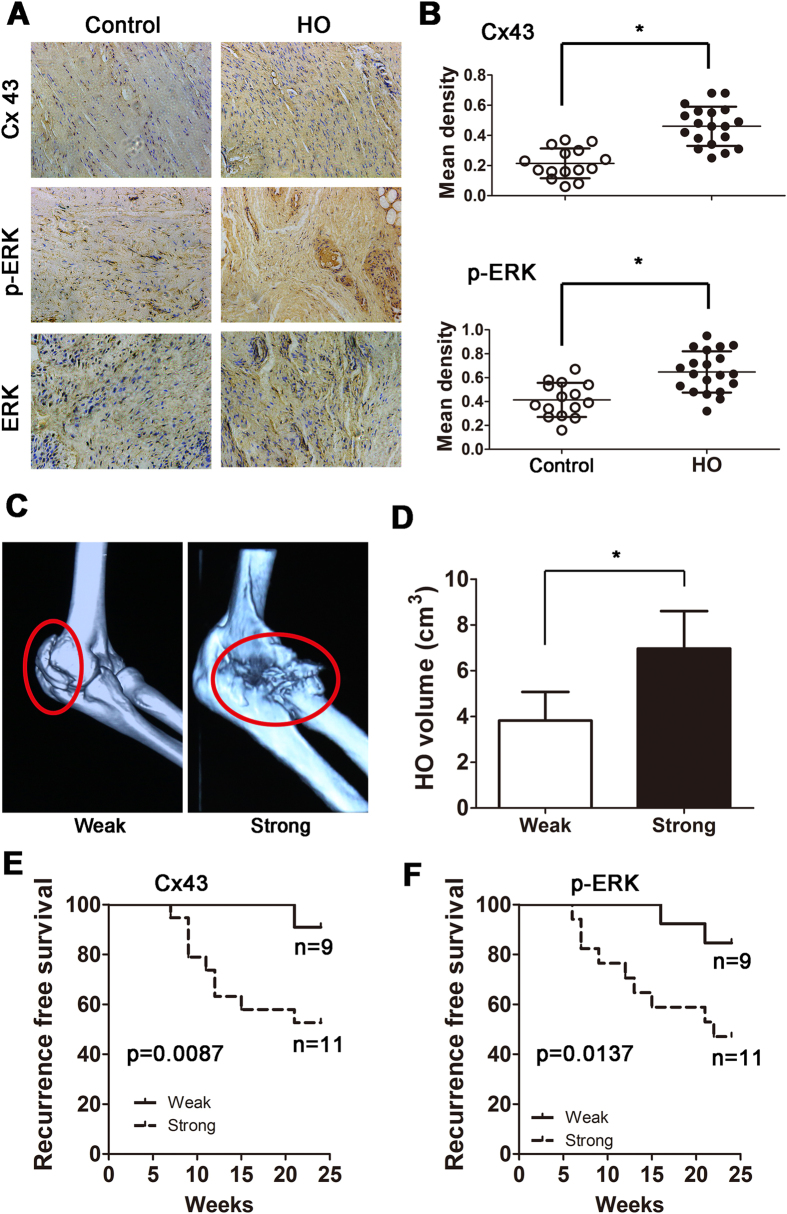
Cx43/ERK signaling is critical for clinical outcomes in traumatic HO patients. (**A**) Normal bones (n = 15) and HO tissues (n = 20) were decalcified. Expression of Cx43 and p-ERK was detected by immunohistochemistry. (**B**) Quantification of Cx43 and p-ERK expression. (**C**) A typical radiographic three-dimensional reconstruction CT scan is shown for elbow HO with weak and strong expression of Cx43. (**D**) The volumes of the HO with weak and strong Cx43 expression were quantified. (t-test, *p < 0.05). (**E**) The overall recurrence-free survival of patients with weak and strong expression of Cx43 is shown. (**F**) The overall recurrence-free survival of patients with weak and strong expression of p-ERK is shown. (log-rank test).

To verify the effects of Cx43/ERK signaling on clinical outcomes, HO lesions were resected from elbow trauma patients (n = 20), and bones from amputation patients were used as a control (n = 15). All bones were demineralized and subjected to an immunohistochemical assay. We observed that HO tissues exhibited increased Cx43 and p-ERK expressions (Fig. 6A,B). According to the Cx43 and p-ERK staining density, the HO patients were divided into weak (<0.5, n = 9) and strong (>0.5, n = 11) groups. The 3D CT showed that strong expression of Cx43 was correlated with increased HO volume (Fig. 6C,D; p = 0.016). Furthermore, we observed that HO patients with strong Cx43 expression showed a higher recurrence rate after surgical treatment (Fig. 6E). Similar results were observed in the strong p-ERK HO patients (Fig. 6F). In addition, tissues from the mice showed that Cx43 was highly expressed in endochondral bone and HO (Supplementary Fig. 8A). Soft tissue around the recurrent HO from the patients exhibited a higher Cx43 baseline than soft tissue around the non-recurrent HO (Supplementary Fig. 8B).

## Discussion

HO is a pathologic process in patients with severe musculoskeletal trauma and burns and with genetic mutations that confer hyperactive ossification. Although the exact mechanism of ectopic bone formation is still unknown, progenitor cells capable of osteogenic differentiation are known to play an essential role^14^. In this study, we leveraged our knowledge to demonstrate that Cx43/ERK signaling represents a route for traumatic ectopic bone formation. The present study demonstrates that Cx43/ERK might be essential for ectopic bone formation in a mouse model, which is highly consistent with our clinical observations. These findings suggest that targeting Cx43/ERK pathway may represent a solution for traumatic HO.

Previous studies have elucidated the genetic basis of HO as fibrodysplasia ossificans progressiva (FOP), a genetic disease that causes progressive HO. Genetic mutations in the ACVR1 gene conferring hyperactivity is regarded as the most important cause for FOP^15^,^16^. Although the genetic basis of FOP has been known for several years, the identity of the precursor cells that actually differentiate along the osteogenic lineage to form HO remain unclear^17^. Several studies have demonstrated that vascular endothelial cells could be a candidate for the cellular origin of HO^18^,^19^, while other studies provide strong evidence that tissue-resident MSCs or progenitor cell populations are the original cells in HO^20^,^21^. In trauma and wounds, MSCs in the tissue and circulation contribute to wound and fracture healing, which requires osteogenic differentiation of MSCs^22^. A recent study showed that activated matrix metalloproteinase 9 contributes to HO^23^. Agarwal S *et al.* found that pharmacologic inhibition of Hif1α abolished HO formation^21^.

Gap junctions, which are specialized intercellular membrane channels, are highly dynamic structures that are regulated by kinase-mediated signaling pathways and interactions with other proteins^24^. In the bone tissue, the most abundantly expressed gap junction protein is Cx43, which is expressed in osteoblasts, osteocytes and osteoclasts^25^. Previous studies have shown that alterations in Cx43 expression or function modulate the expression of osteoblast genes^26^,^27^. It has been shown that Cx43 expression is essential for normal osteoblastic gene expression and function in osteoblastic cell lines^28^. Thi *et al.* demonstrated that mineralization is impaired in osteoblastic cells isolated from Cx43 knockout mice^29^. In addition, osteogenic differentiation markers including Runx2, osteocalcin (OCN), collagen 1 and BSP are decreased in osteoblastic cells isolated from Cx43 knockout mice and in osteoblast cells overexpressing Cx45, which acts as an antagonist for Cx43^30^,^31^. Evans KN *et al.* showed that multiple osteogenesis-related genes are upregulated in traumatic HO^32^. These studies suggested that Cx43 might play an important role in HO formation. Using a model of trauma-induced HO, we provided evidence that overexpression of Cx43 plays an important role in the development of HO, in which Cx43 exerts its effects by promoting the expression of the osteogenic markers.

Endochondral ossification is a common process in HO formation^33^. Previous studies showed that activation of β-catenin stimulates endochondral ossification^34^. Consistent with their findings, we observed upregulated Wnt-3a expression in the gene chip. These data suggest that Wnt/β-catenin may play a role in HO formation. Moreover, our histologic analysis showed that Cx43 inhibition decreased early endochondral ossification. However, once the HO was completely developed, inhibition of Cx43 could not decrease the mature ectopic bones. These data suggest that inhibition of Cx43 at the early stage of HO may be benefit for the treatment of HO. Additionally, we demonstrated that HO patients with lower expression of Cx43 exhibit less recurrence after the surgical resection. These results suggested that Cx43 base line may affect the primary and recurrent HO formation.

Cx43-mediated regulation of osteoblast differentiation and gene expression involves transcription factors Runx2 and Osterix (Sp7), both of which are master regulators of osteogenesis^35^. Disruption of the Cx43 channels by overexpression of Cx45 or by specific inhibitors in osteoblast cells decreases activation of ERK signaling^36^,^37^. Moreover, decreased ERK activity leads to reduced phosphorylation and DNA binding to the transcription factors, resulting in reduced transcription of the osteogenic marker genes^38^,^39^. Similarly, our data showed that ERK signaling was activated and that Runx2 expression was enhanced in the HO tissues. Our findings indicate that Cx43 and osteogenic medium have different effects on ERK activation. On one hand, p-ERK expression was enhanced during osteogenesis, but on the other hand, inhibition of Cx43 decreased the ERK activation. Although Cx43 shRNA did not completely abolish the p-ERK expression, it maintained the p-ERK expression at the un-differentiated level and inhibited osteoblast differentiation. These data indicated that a decreased or no changed p-ERK expression would not induce osteogenesis. Although inhibition of Cx43 or ERK signaling decreased the formation of HO (approximately 50%), it could not completely prevent the HO. Therefore, surgical resection of the ectopic bones is necessary in some cases. Moreover, inhibition of Cx43 would result in delayed fracture healing, impaired angiogenesis and tissue regeneration^7^. These observations suggest that application of Cx43 in HO treatment may cause disorders in other organs/tissues. Thus the clinical application of Cx43 would be limited under certain situations.

In summary, we have identified an important mechanism that may determine the progression and development of traumatic HO. We show that increased expression of Cx43 enhances the transcription of Runx2 and osteogenic differentiation in a manner that promotes HO progression. In examining the routes of Cx43-dependent activation of ERK, we have demonstrated the relevant function of Cx43/ERK signaling in HO development. We have demonstrated that pharmacotherapy with Cx43 or ERK inhibitors, such as shRNA or U0126, can markedly reduce extraskeletal bone formation in a traumatic model of HO. Using patient data and human HO specimens, we confirmed that elevated levels of Cx43 and p-ERK increase the risk of recurrence after surgical resection. The results reveal a new mechanism of HO formation and identify a potential therapeutic target.

## Materials and Methods

### Patients and specimens

Twenty patients who underwent surgical resection for elbow lesions between January 2010 and December 2015 were evaluated in this study, and the control bones were collected from 15 patients who received traumatic amputation (tibia, femur, radius and ulna). The patients, with a median age of 32 years (range: 21–62 years), were healthy without metabolic, inherited or other diseases that may affect the current study. No significant difference was observed in composition regarding age or sex in these two groups (p > 0. 05). The mature HO lesions were removed by surgery 8–10 months after the injury. The control bones from the amputation patients were collected 2–4 hours after the injury during surgery. After surgery, all the patients were examined with x-rays or CT monthly, and the follow-up time was 25 weeks. Approval for this study was obtained from the ethics committee of Shanghai Jiao Tong University Affiliated Sixth People’s Hospital, and written informed consent was obtained from the patients or their legal guardians. The experiment was performed in accordance with approved guidelines.

### RNA isolation and gene expression profiling

Soft tissues and epiphyses were removed, epiphyses were cut off, and diaphyses were flushed with phosphate-buffered saline solution (PBS) to remove bone marrow and blood. All the samples were shock-frozen in liquid nitrogen, pulverized, and dissolved in Trizol (Life Technologies, Carlsbad, CA, USA). Total RNA was isolated from all bone or HO samples using a Trizol isolation kit according to the manufacturer’s instructions. Quantity and quality measurements were carried out using a NanoDrop ND-1000 spectrophotometer (NanoDrop Technologies, Wilmington, DE, USA) and an Agilent 2100 Bioanalyzer (Agilent Technologies, Santa Clara, California, USA). All samples were then subjected to downstream genome-wide microarray analysis. All of the hybridization experiments were performed using Affymetrix HG-U133 Plus 2.0 GeneChips according to the manufacturer’s recommendations. The raw data were normalized using Genespring GX11 Software (Agilent Technologies, Santa Clara, CA, USA) with default parameters (MAS5 Summarization Algorithm, median of all samples as baseline transformation).

### Cell Culture

The preosteoblast cells MC-3T3 and C2C12 skeletal muscle cells were obtained from the Chinese Academy of Sciences (Shanghai, China). Cells were cultured in α-MEM (1000 mg/L glucose) with 10% fetal bovine serum (FBS). After confluency was reached, the culture medium was changed to an osteogenic differentiation medium (OM), which contained 10% FBS, 50 μM L-ascorbic acid, 10 mM glycerol-2-phosphate, and 100 nM dexamethasone.

### shRNA transfection

MC-3T3 cells were transfected with lentiviral particles loaded with either small hairpin RNA (shRNA) against Cx43 or ERK (Santa Cruz Biotechnology, Santa Cruz, CA; Cat No. sc-35091–SH for Cx43, Cat No. sc-44206-SH for ERK) or a scrambled control (Santa Cruz Biotechnology, Santa Cruz, CA). The shRNA plasmids consisted of a pool of 3 to 5 lentiviral vector plasmids, each encoding target-specific 19–25 nt shRNAs designed to knockdown gene expression. The control shRNA plasmid encoded a scrambled shRNA sequence designed to not lead to the specific degradation of any known cellular mRNA. The cells were subsequently exposed to 3 μg/ml of puromycin (Sigma, USA) for 1 month. The several available clones were expanded and maintained in 1 μg/ml of puromycin to remove cells without shRNA expression.

### Cell viability analysis

MT-3T3 and C2C12 cells were seeded in 96-well plates at a density of 1 × 10^4^ per well and treated as follows: the culture medium was discarded, and the cells were rinsed 3 times with PBS. Subsequently, the viable cells were quantitated using a cell counting kit-8 (CCK-8, Dojindo, Japan) according to the manufacturer’s instructions. Briefly, the cells were incubated in α-MEM medium containing 10 μl of CCK-8 solution at 37 °C for 2.5 hours. Then, the optical density (OD) at 450 nm was determined using a microplate reader (BIOTEK, Vermont, USA), and the ratio of viable cells was calculated.

### ALP and Alizarin Red S staining

For ALP staining, cells were stained with 5-bromo-4-chloro-3-indolyl-phosphate/nitro-blue tetrazolium solution (Sigma-Aldrich) for 45 minutes at 37 °C to visualize ALP activity. For the Alizarin Red S staining, the cells were washed 3 times with PBS and fixed with 4% paraformaldehyde for 10 min at room temperature. After 3 washes with deionized water, the calcium mineral deposits were stained for 10 min with 2% Alizarin Red S (pH 4.2), rinsed with water, and visualized by light microscopy.

### Quantitative measurement of alkaline phosphatase activity

The cells were washed twice with PBS and lysed with 1% Triton X-100 supplemented with protease and kinase inhibitors, followed by freeze–thaw cycles. ALP activity was assayed using p-nitrophenylphosphate (Sigma-Aldrich) as a substrate. Lysates were incubated with 100 μl of 3 mg/ml substrate solution for 30 min at 37 °C, and the reaction was stopped with 20 mM NaOH. The protein content was measured by BCA (Thermo Scientific, Rockford, IL) according to the manufacturer’s instructions. ALP activity was then expressed as Sigma unit/min/mg of protein.

### Quantitative analysis of mineralization

The calcium deposits from osteoblast cells were washed 3 times with PBS and incubated for 24 h at 4 °C in 0.5 M HCl. Then, the calcium content in the HCl supernatants was measured using the Calcium Colorimetric Assay Kit (BioVision, Mountain View, CA, USA).

### Western blot

The cells were washed in ice-cold PBS before lysis with a cell lysis buffer (Cell Signaling Technology, MA, USA). All samples were clarified by centrifugation at 12,000 rpm for 10 minutes at 4 °C. Then, the protein concentrations were determined using the BCA Protein Assay (Thermo Scientific, IL, USA). Equal amounts of total protein lysates were separated by SDS-PAGE, and bands were transferred to a nitrocellulose membrane. The membranes were blotted with the following primary antibodies: p-ERK (Cell Signaling Technology, Cat No. 4370, 42 kD), ERK (Cell Signaling Technology, Cat No. 9102, 42 kD), β-actin (Cell Signaling Technology, Cat No. 4970, 45 kD), Cx43 (Abcam, Cat No. ab11370, 43 kD), Runx2 (Abcam, Cat No. ab23981, 57 kD) and BSP (Abcam, Cat No. ab125227, 35 kD). Bound antibodies were detected with an Odyssey Infrared Imaging System (LI-COR Biosciences, Lincoln, NE, USA). Densitometric analysis of the protein bands was performed with Image-Pro Plus 4.5 software (Media Cybernetics, Silver Spring, MD).

### RNA isolation and quantitative real-time PCR

Total RNA was prepared using Qiagen RNeasy Mini Kit (Qiagen, Valencia, CA, USA) for cellular extracts. cDNA was then generated from 1 μg of RNA using the iScript cDNA Synthesis Kit (Bio-Rad, Hercules, CA) according to the manufacturer’s instructions. Quantitative real-time expression analysis was performed using an ABI 7500 Sequencing Detection System and SYBR Premix Ex Taq (Takara, Japan). Relative expression of mRNA was determined after normalization using the ΔCt method. The following primers were used: GAPDH, 5′-ATGGGGAAGGTGAAGGTCG-3′ (forward) and 5′-GGGGTCATTGATGGCAACAATA-3′ (reverse); Runx2, 5′-CCGCCTCAGTGATTTAGGGC-3′ (forward) and 5′-GGGTCTGTAATCTGACTCTGTCC-3′ (reverse); ALP, 5′-TGAGGGTGTGGCTTACCAG-3′ (forward) and 5′-GATGGACGTGTAGGCTTTGCT-3′ (reverse); BSP, 5′-CAGGGAGGCAGTGACTCTTC-3′ (forward) and 5′-AGTGTGGAAAGTGTGGCGTT-3′ (reverse); and Col-1, 5′-GAAAAGGGTACATCGGGTGAG-3′ (forward) and 5′-GAACCCATCGAGTCCTGGT-3′ (reverse).

### Mice

The animal experimental protocols were approved by the Animal Research Committee of Shanghai Jiao Tong University Affiliated Sixth People’s Hospital. The experiment was performed in accordance with approved guidelines. Four-week-old male BALB/c mice received an Achilles tenotomy (n = 12 per group) with sharp dissection at the midpoint in the left leg. A 1-cm incision was made on the lateral aspect of the Achilles tendon with a surgical knife. Subsequently, the Achilles tendon was exposed from its origin on the distal end of the gastrocnemius to the insertion at the calcaneus. The Achilles tendon was then divided sharply at its midpoint. The incision was then closed with absorbable sutures. Then, mice received an injection of 2 μg of Cx43 shRNA complexed with 2 μl of Lipo 2000 dissolved in 10 μl of α-MEM. Sham mice received an injection of 2 μg of scrambled shRNA plasmid complexed with 2 μl of Lipo 2000 as a negative control. All mice were treated weekly with an shRNA or scrambled shRNA local injection in the lesion in the tendon. For the *in vivo* p-ERK inhibition experiment, mice were treated by local injection of U0126 (Calbiochem, CA, USA; 100 μg/mouse) in the lesion in the tendon every 3 days, and DMSO was used as a negative control. The mice were maintained for 8 weeks, and the injured limbs were harvested after euthanasia.

### Micro-CT and x-ray analysis

The left tibias dissected from mice were fixed with 4% paraformaldehyde for 24 h, then scanned and analyzed with a SkyScan microcomputed tomography system (Kontich, Belgium) with a 9-μm pixel size. The X-ray voltage and current were set to 80 kV and 80 μA, respectively. The region of interest (ROI) was defined to include the entire tibia, and two-dimensional (2D) image stacks were visually inspected to ensure that all heterotopic bone was included within the ROI. The Nrecon reconstruction software (NRecon v.1.4.4, SkyScan) was used to create 2-D images. For the reconstruction parameters, ring artifact correction and smoothing were fixed at zero and the beam hardening correction was set at 0%. The contrast limits were applied following the manufacture’s instructions. The lower limit was zero and the upper limit was at the top end of the brightness spectrum. Following the reconstruction, the volume of interest (VOI) was selected within the reconstructed images to calibrate the standard unit of X-ray computed tomography density (Hounsfield unit, HU) using CTAn analysis software (v.1.6.0, SkyScan). The HU values of two BMD phantom rods were measured and converted from HU to BMD values (g/cm3). The bone volume of HO was also analyzed using CTAn. Analysis of x-ray was performed with a image-pro plus 4.5 software (Media Cybernetics, Silver Spring, MD). The HO area was calculate and normalized to the control group.

### Histology

At 8 weeks post-tenotomy, animals were euthanized, and the tenotomized limbs were fixed in 4% paraformaldehyde. HO tissues from patients were collected in surgical operations. All of these specimens were decalcified in a 10% EDTA solution for 1 month, were embedded in paraffin and were cut into 5-μm sections for staining. Immunohistochemical staining was carried out with primary antibodies against Cx43 (Abcam, Cat No. ab11370), p-ERK (Cell Signaling Technology, Cat No. 4370), ERK (Cell Signaling Technology, Cat No. 9102), Runx2 (Abcam, Cat No. ab23981) and BSP (Abcam, Cat No. ab125227) with a 1:1,000 dilution of an appropriate secondary antibody. Protein expression was visualized with a DakoCytomation Envision staining kit. Sections were stained with 0.1% Safranin-O and 0.02% Fast Green (Sigma-Aldrich, Oakville, ON, Canada) according to the manufacturer’s instructions. The mean density of the positive area was measured by Image Pro Plus 6.0 (IPP) image analysis software. Three random slides were selected, and five random fields of images per sample were taken.

### Statistical analyses

The data are represented as the mean ± standard deviation (SD). Comparisons between groups were performed using Student’s t-test, and one-way ANOVA was used for multiple comparisons. Recurrence-free survival rates were compared using Kaplan–Meier survival curves. Statistical significance was set at p < 0.05.

## Additional Information

**How to cite this article**: Tu, B. *et al.* Inhibition of connexin 43 prevents trauma-induced heterotopic ossification. *Sci. Rep.* **6**, 37184; doi: 10.1038/srep37184 (2016).

**Publisher’s note**: Springer Nature remains neutral with regard to jurisdictional claims in published maps and institutional affiliations.

## Supplementary Material

Supplementary Information

## References

[b1] Nauth A. *et al.* Heterotopic ossification in orthopaedic trauma. J Orthop Trauma 26, 684–688 (2012). 23010648 10.1097/BOT.0b013e3182724624 PMC3504617

[b2] Potter B. K. *et al.* Heterotopic ossification following combat-related trauma. J Bone Joint Surg Am 92 Suppl 2, 74–89 (2010). 21123594 10.2106/JBJS.J.00776

[b3] Kaplan F. S., Glaser D. L., Hebela N. & Shore E. M. Heterotopic ossification. J Am Acad Orthop Surg 12, 116–125 (2004). 15089085 10.5435/00124635-200403000-00007

[b4] Jackson W. M., Aragon A. B., Bulken-Hoover J. D., Nesti L. J. & Tuan R. S. Putative heterotopic ossification progenitor cells derived from traumatized muscle. J Orthop Res 27, 1645–1651 (2009). 19517576 10.1002/jor.20924 PMC3014572

[b5] Downey J. *et al.* Prospective heterotopic ossification progenitors in adult human skeletal muscle. Bone 71, 164–170 (2015). 25445454 10.1016/j.bone.2014.10.020

[b6] Xu H. *et al.* Connexin 43 channels are essential for normal bone structure and osteocyte viability. J Bone Miner Res 30, 436–448 (2015). 25270829 10.1002/jbmr.2374 PMC4333056

[b7] Plotkin L. I. & Bellido T. Beyond gap junctions: Connexin43 and bone cell signaling. Bone 52, 157–166 (2013). 23041511 10.1016/j.bone.2012.09.030 PMC3513515

[b8] Loiselle A. E., Lloyd S. A., Paul E. M., Lewis G. S. & Donahue H. J. Inhibition of GSK-3beta rescues the impairments in bone formation and mechanical properties associated with fracture healing in osteoblast selective connexin 43 deficient mice. PLoS One 8, e81399 (2013). 24260576 10.1371/journal.pone.0081399 PMC3832658

[b9] Loiselle A. E., Paul E. M., Lewis G. S. & Donahue H. J. Osteoblast and osteocyte-specific loss of Connexin43 results in delayed bone formation and healing during murine fracture healing. J Orthop Res 31, 147–154 (2013). 22718243 10.1002/jor.22178 PMC3640531

[b10] Niger C. *et al.* The regulation of runt-related transcription factor 2 by fibroblast growth factor-2 and connexin43 requires the inositol polyphosphate/protein kinase Cdelta cascade. J Bone Miner Res 28, 1468–1477 (2013). 23322705 10.1002/jbmr.1867 PMC3657330

[b11] Kim H. J., Kim J. H., Bae S. C., Choi J. Y. & Ryoo H. M. The protein kinase C pathway plays a central role in the fibroblast growth factor-stimulated expression and transactivation activity of Runx2. J Biol Chem 278, 319–326 (2003). 12403780 10.1074/jbc.M203750200

[b12] Park O. J., Kim H. J., Woo K. M., Baek J. H. & Ryoo H. M. FGF2-activated ERK mitogen-activated protein kinase enhances Runx2 acetylation and stabilization. J Biol Chem 285, 3568–3574 (2010). 20007706 10.1074/jbc.M109.055053 PMC2823497

[b13] Lecanda F. *et al.* Connexin43 deficiency causes delayed ossification, craniofacial abnormalities, and osteoblast dysfunction. J Cell Biol 151, 931–944 (2000). 11076975 10.1083/jcb.151.4.931 PMC2169447

[b14] Peterson J. R. *et al.* Effects of aging on osteogenic response and heterotopic ossification following burn injury in mice. Stem Cells Dev 24, 205–213 (2015). 25122460 10.1089/scd.2014.0291 PMC4291203

[b15] Shore E. M. *et al.* A recurrent mutation in the BMP type I receptor ACVR1 causes inherited and sporadic fibrodysplasia ossificans progressiva. Nat Genet 38, 525–527 (2006). 16642017 10.1038/ng1783

[b16] Kaplan F. S., Chakkalakal S. A. & Shore E. M. Fibrodysplasia ossificans progressiva: mechanisms and models of skeletal metamorphosis. Dis Model Mech 5, 756–762 (2012). 23115204 10.1242/dmm.010280 PMC3484858

[b17] Kan L. & Kessler J. A. Evaluation of the cellular origins of heterotopic ossification. Orthopedics 37, 329–340 (2014). 24810815 10.3928/01477447-20140430-07

[b18] Medici D. *et al.* Conversion of vascular endothelial cells into multipotent stem-like cells. Nat Med 16, 1400–1406 (2010). 21102460 10.1038/nm.2252 PMC3209716

[b19] Lounev V. Y. *et al.* Identification of progenitor cells that contribute to heterotopic skeletogenesis. J Bone Joint Surg Am 91, 652–663 (2009). 19255227 10.2106/JBJS.H.01177 PMC2663346

[b20] Wosczyna M. N., Biswas A. A., Cogswell C. A. & Goldhamer D. J. Multipotent progenitors resident in the skeletal muscle interstitium exhibit robust BMP-dependent osteogenic activity and mediate heterotopic ossification. J Bone Miner Res 27, 1004–1017 (2012). 22307978 10.1002/jbmr.1562 PMC3361573

[b21] Agarwal S. *et al.* Inhibition of Hif1alpha prevents both trauma-induced and genetic heterotopic ossification. Proc Natl Acad Sci USA 113, E338–E347 (2016). 26721400 10.1073/pnas.1515397113 PMC4725488

[b22] Jones E. & Yang X. Mesenchymal stem cells and bone regeneration: current status. Injury 42, 562–568 (2011). 21489533 10.1016/j.injury.2011.03.030

[b23] Davis E. L. *et al.* Location-dependent heterotopic ossification in the rat model: The role of activated matrix metalloproteinase 9. J Orthop Res (2016). 10.1002/jor.23216 PMC5001934 26919547

[b24] Matsuuchi L. & Naus C. C. Gap junction proteins on the move: connexins, the cytoskeleton and migration. Biochim Biophys Acta 1828, 94–108 (2013). 22613178 10.1016/j.bbamem.2012.05.014

[b25] Yellowley C. E., Li Z., Zhou Z., Jacobs C. R. & Donahue H. J. Functional gap junctions between osteocytic and osteoblastic cells. J Bone Miner Res 15, 209–217 (2000). 10703922 10.1359/jbmr.2000.15.2.209

[b26] Li Z., Zhou Z., Saunders M. M. & Donahue H. J. Modulation of connexin43 alters expression of osteoblastic differentiation markers. Am J Physiol Cell Physiol 290, C1248–C1255 (2006). 16319124 10.1152/ajpcell.00428.2005

[b27] Lima F., Niger C., Hebert C. & Stains J. P. Connexin43 potentiates osteoblast responsiveness to fibroblast growth factor 2 via a protein kinase C-delta/Runx2-dependent mechanism. Mol Biol Cell 20, 2697–2708 (2009). 19339281 10.1091/mbc.E08-10-1079 PMC2688549

[b28] Chung D. J. *et al.* Low peak bone mass and attenuated anabolic response to parathyroid hormone in mice with an osteoblast-specific deletion of connexin43. J Cell Sci 119, 4187–4198 (2006). 16984976 10.1242/jcs.03162

[b29] Thi M. M., Urban-Maldonado M., Spray D. C. & Suadicani S. O. Characterization of hTERT-immortalized osteoblast cell lines generated from wild-type and connexin43-null mouse calvaria. Am J Physiol Cell Physiol 299, C994–C1006 (2010). 20686067 10.1152/ajpcell.00544.2009 PMC2980299

[b30] Stains J. P., Lecanda F., Screen J., Towler D. A. & Civitelli R. Gap junctional communication modulates gene transcription by altering the recruitment of Sp1 and Sp3 to connexin-response elements in osteoblast promoters. J Biol Chem 278, 24377–24387 (2003). 12700237 10.1074/jbc.M212554200

[b31] Stains J. P. & Civitelli R. Gap junctions regulate extracellular signal-regulated kinase signaling to affect gene transcription. Mol Biol Cell 16, 64–72 (2005). 15525679 10.1091/mbc.E04-04-0339 PMC539152

[b32] Evans K. N. *et al.* Osteogenic gene expression correlates with development of heterotopic ossification in war wounds. Clin Orthop Relat Res 472, 396–404 (2014). 24136804 10.1007/s11999-013-3325-8 PMC3890153

[b33] Huegel J., Enomoto-Iwamoto M., Sgariglia F., Koyama E. & Pacifici M. Heparanase stimulates chondrogenesis and is up-regulated in human ectopic cartilage: a mechanism possibly involved in hereditary multiple exostoses. Am J Pathol 185, 1676–1685 (2015). 25863260 10.1016/j.ajpath.2015.02.014 PMC4450318

[b34] Kitagaki J. *et al.* Activation of beta-catenin-LEF/TCF signal pathway in chondrocytes stimulates ectopic endochondral ossification. Osteoarthritis Cartilage 11, 36–43 (2003). 12505485 10.1053/joca.2002.0863

[b35] Lecanda F. *et al.* Gap junctional communication modulates gene expression in osteoblastic cells. Mol Biol Cell 9, 2249–2258 (1998). 9693379 10.1091/mbc.9.8.2249 PMC25477

[b36] Yamada A. *et al.* Connexin 43 Is Necessary for Salivary Gland Branching Morphogenesis and FGF10-induced ERK1/2 Phosphorylation. J Biol Chem 291, 904–912 (2016). 26565022 10.1074/jbc.M115.674663 PMC4705408

[b37] Buo A. M. & Stains J. P. Gap junctional regulation of signal transduction in bone cells. FEBS Lett 588, 1315–1321 (2014). 24486014 10.1016/j.febslet.2014.01.025 PMC3989400

[b38] Niger C. *et al.* ERK acts in parallel to PKCdelta to mediate the connexin43-dependent potentiation of Runx2 activity by FGF2 in MC3T3 osteoblasts. Am J Physiol Cell Physiol 302, C1035–C1044 (2012). 22277757 10.1152/ajpcell.00262.2011 PMC3330735

[b39] Jeong H. M. *et al.* Risedronate increases osteoblastic differentiation and function through connexin43. Biochem Biophys Res Commun 432, 152–156 (2013). 23376077 10.1016/j.bbrc.2013.01.068

