# Supplementary material for: Inhibition of connexin 43 prevents trauma-induced heterotopic ossification
Source: Sci Rep. 2016 Nov 16;6:37184. doi: 10.1038/srep37184 (PMC5111117; doi:10.1038/srep37184)
Supplement: Supplementary Information [file srep37184-s1.pdf]

Inhibition of connexin 43 prevents trauma-induced heterotopic ossification

Bing Tu[a,#]

Shen Liu[a,#]

Guangwang Liu[b,#]

Zhiwei Li[a]

Yangbai Sun[a]

Cunyi Fan[a] *

[a] Department of Orthopaedic Surgery, Shanghai Jiaotong University Affiliated Sixth People's Hospital, No. 600 Yishan Road, Shanghai, 200233, China.

[b] Department of Orthopaedic Surgery, the Central Hospital of Xuzhou, Xuzhou Clinical School of Xuzhou Medical College, Xuzhou Hospital (affiliated with Medical College of Southeast University), Jiangsu, 221009, China

[#] These authors contribute equally to this work.

*** Corresponding author. Mailing address: Department of Orthopaedic Surgery, Shanghai Jiaotong University Affiliated Sixth People's Hospital, No. 600 Yishan Road, Shanghai, 200233, China. Tel.: +86-21-6436-9181; Fax: +86-21-6406-3802 E-mail: fancunyi888@163.com.**

Supplementary Information

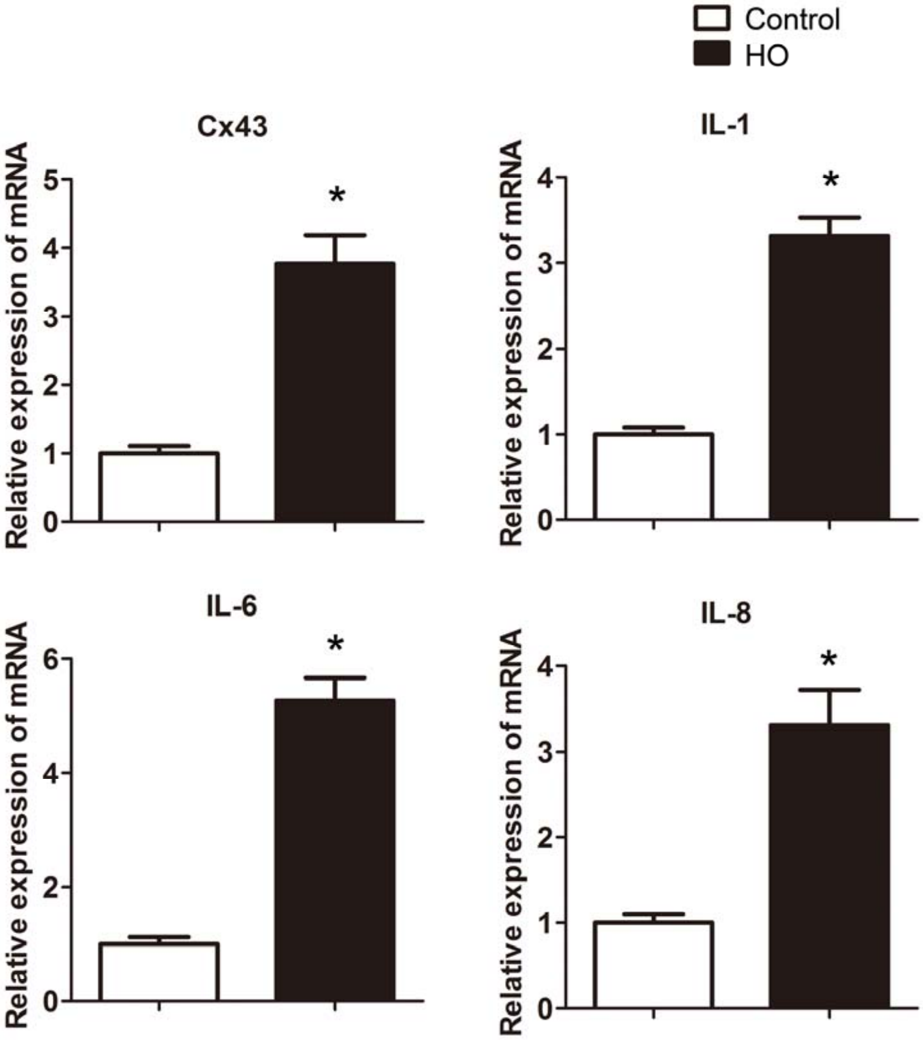

Supplementary Fig. 1. Expression of Cx43 and inflammatory factors is increased in soft tissues around the HO. Soft tissue (joint capsule and ligament) near the HO was collected in the HO surgical resection. Soft tissue (joint capsule and ligament) around the joint of the traumatic amputation patients was used as the control. Total RNA was extracted from these tissues and the expression of Cx43, IL-1, IL-6 and IL-8 mRNA was detected by real-time PCR. (n=5, * $p < 0.05$).

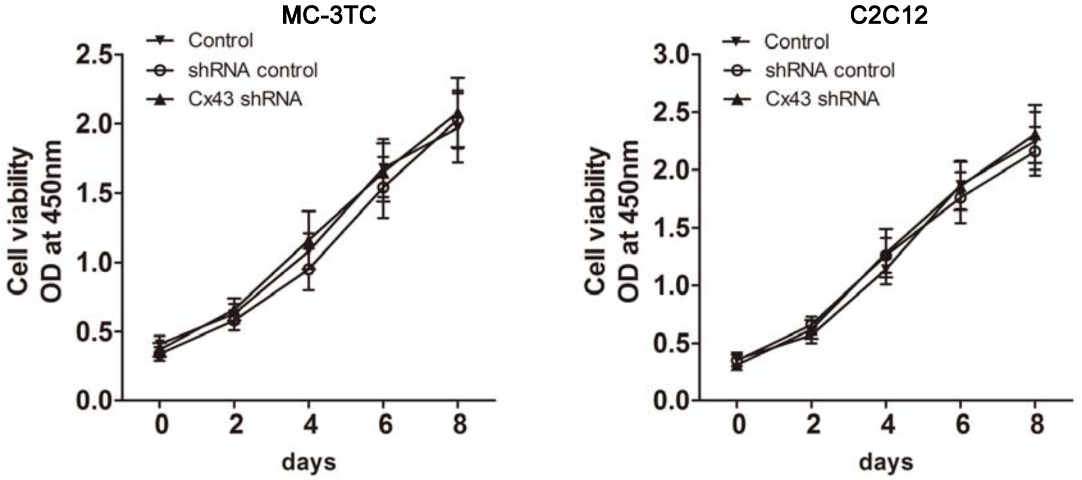

Supplementary Fig. 2. Cx43 shRNA transfection has no effects on the viability of MC-3TC and C2C12 cells. MC-3TC (left) or C2C12 (right) cells were seeded in 96-well plates (1×10^4/well) and transfected with Cx43 shRNA or a control shRNA and cultured for 8 days. The number of viable cells was tested by the CCK-8 assay. ($n=3$, $p>0.05$).

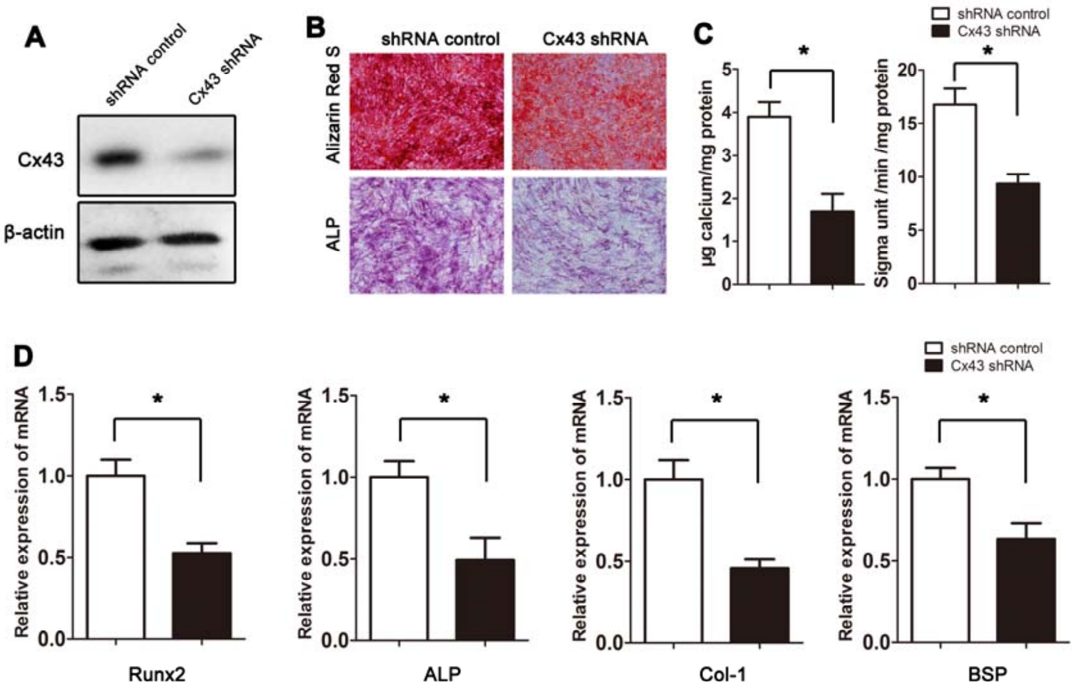

Supplementary Fig. 3. Cx43 promotes MSC osteogenic differentiation. The bone marrow was flushed from femora of 4-week-old mice. The third-passage MSCs were subjected to induction of osteogenic differentiation. (A) Small hairpin RNA (shRNA) was used to knock down Cx43 expression in mice MSCs and a scrambled sequence was used as a control. The expression of Cx43 was detected by Western blot. (B) MSCs transfected with Cx43 shRNA were induced in osteogenic medium. Alizarin Red S (week 2) and ALP (week 1) staining was performed. (C) Calcium deposits and ALP activity were quantified. (D) mRNA expression of osteogenic marker genes was detected by real-time PCR. (n=3, * $p < 0.05$).

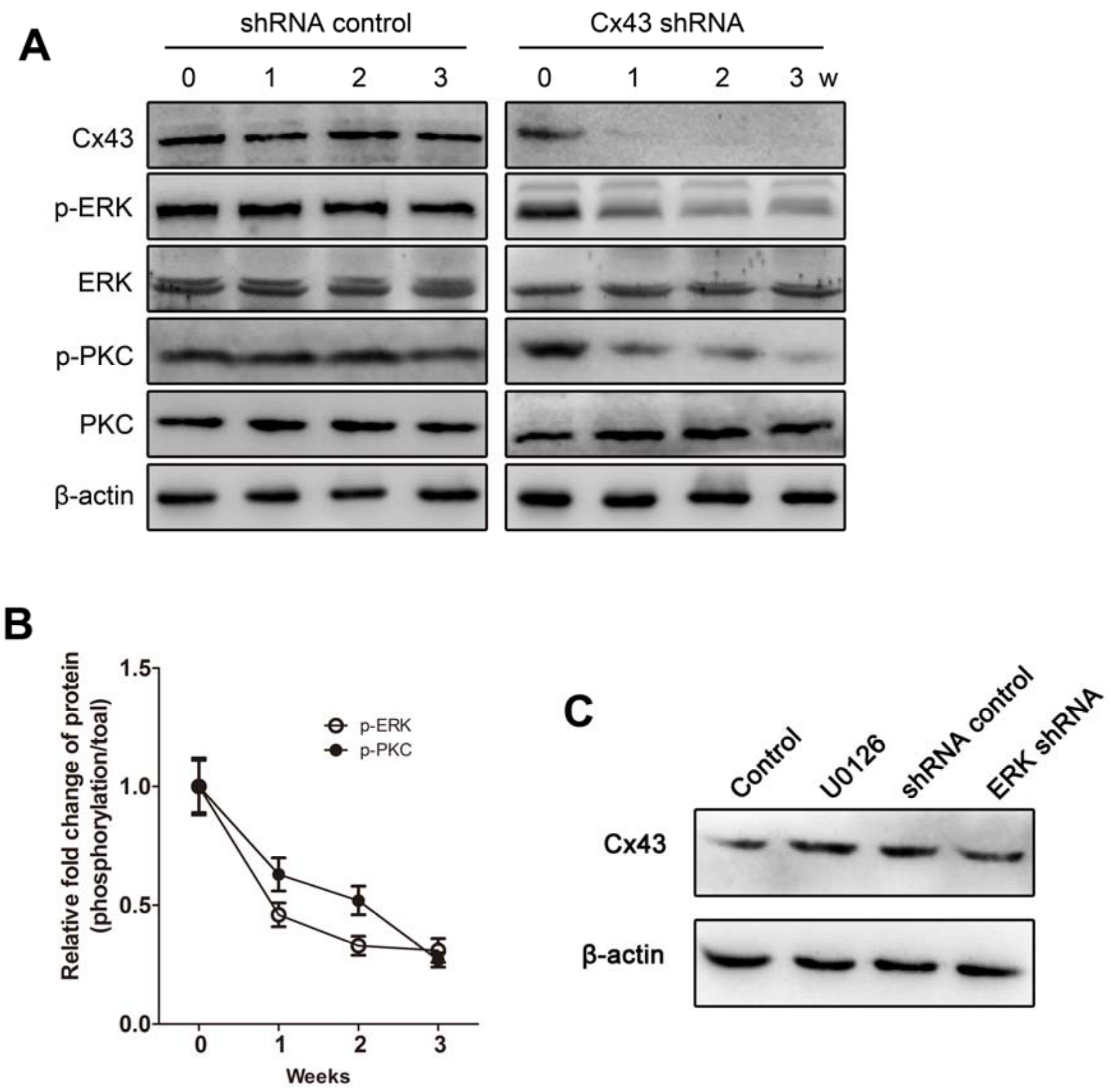

Supplementary Fig. 4. Cx43 knockdown inhibits the activation of ERK and PKC pathway. (A) MC-3T3 cells were transfected with Cx43 shRNA. Cx43, p-ERK, ERK, p-PKC and PKC levels were examined by Western blot at 0, 1, 2 and 3 weeks. (B) Quantification of p-ERK and p-PKC expression. (C) MC-3T3 cells were treated with U0126 or transfected with ERK shRNA for 48 h. The Cx43 expression level was examined by Western blot.

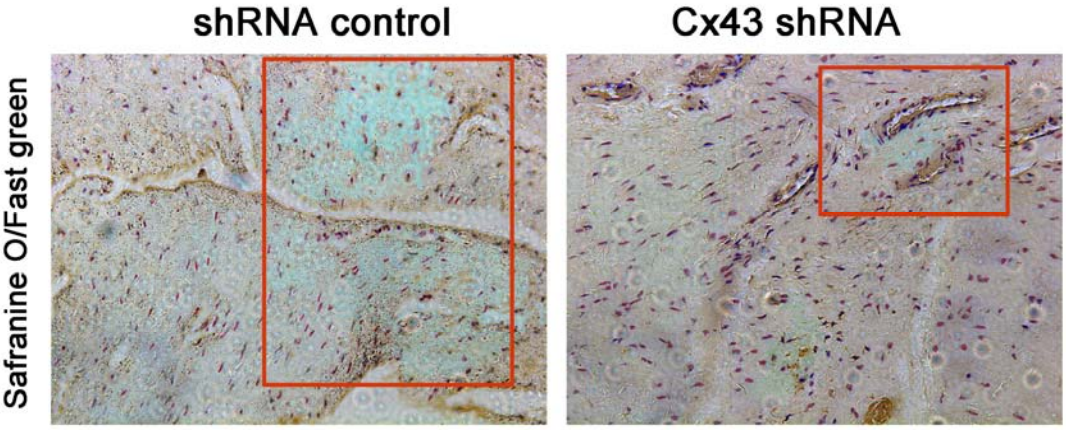

Supplementary Fig. 5. Inhibition of Cx43 decreases the endochondral ossification of HO. Tenotomy mice were treated with local injection of shRNA control or Cx43 shRNA. Safranine O/Fast green staining images demonstrate reduced endochondral ossification in mice treated with Cx43 shRNA at 3 weeks.

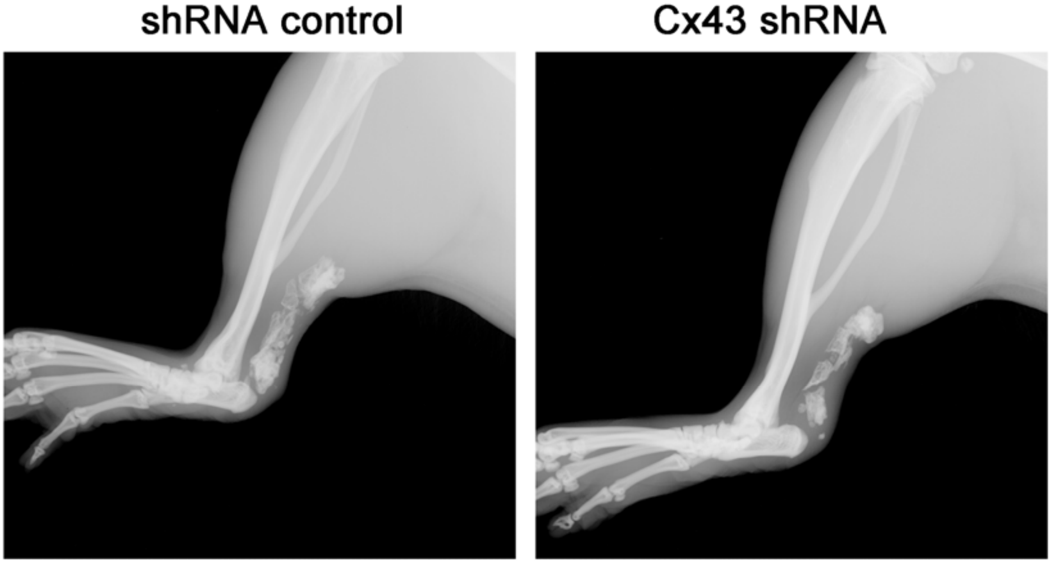

Supplementary Fig. 6. Inhibition of Cx43 has no effect on the developed HO. Tenotomy mice were maintained for 8 weeks to form HO. Then, the mice were treated with a shRNA control or Cx43 shRNA injection. HO was visualized by a radiograph analysis at week 12.

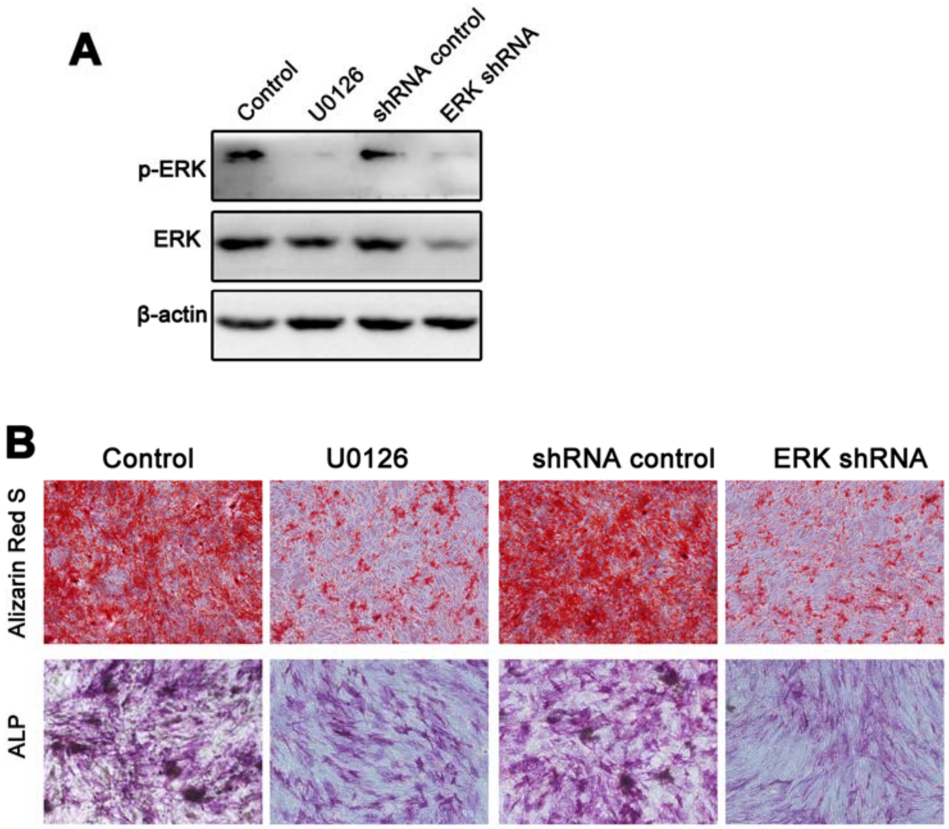

Supplementary Fig. 7. Inhibition of ERK signaling suppressed the osteogenic differentiation of MC-3T3 cells. (A) MC-3T3 cells treated with U0126 or transfected with ERK shRNA were induced in osteogenic medium. The expression of p-ERK and ERK was verified by Western blot. (B) Alizarin Red S (week 2) and ALP (week 1) staining was performed.

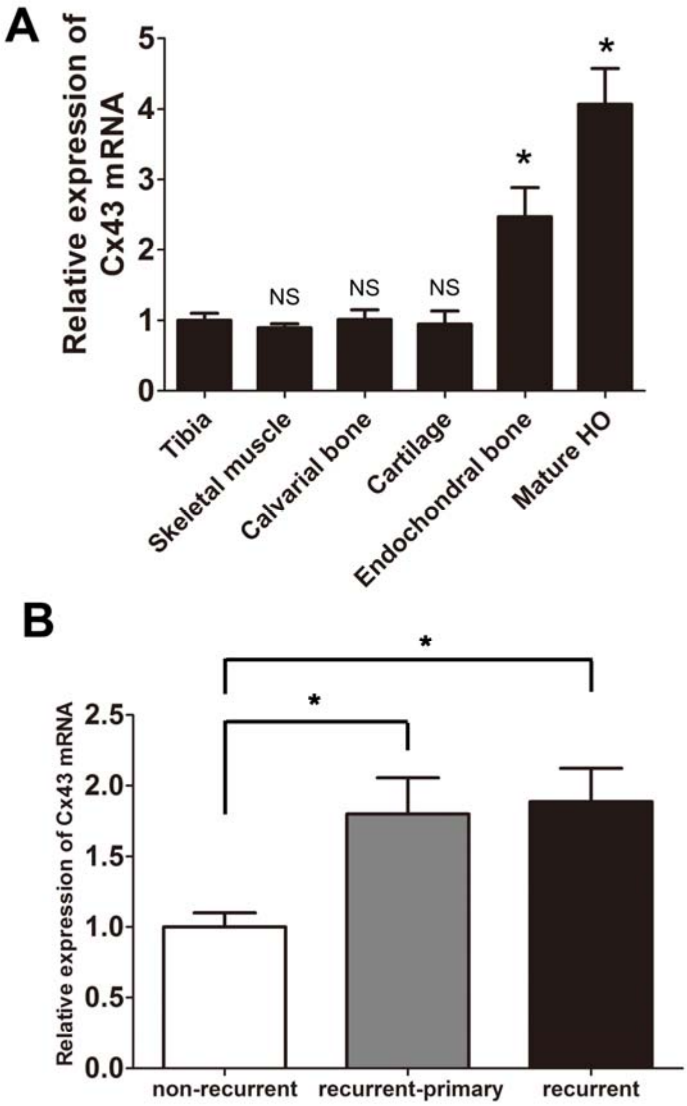

Supplementary Fig. 8. Cx43 expression in bone, cartilage, primary and recurrent HO tissues. (A) The tibia, skeletal muscle, calvarial bone, cartilage, endochondral bone (3 weeks HO at early endochondral ossification stage) and mature HO (8 weeks HO) in mice were collected. Cx43 mRNA expression was detected by real-time PCR. (B) Soft tissues (joint capsule and ligament) near the primary non-recurrent HO and recurrent HO (including primary and recurrent samples) of the patients were collected in the surgical resection. Total RNA was extracted from these tissues and the expression of Cx43 was detected by real-time PCR. (n=5, * $p < 0.05$).
